# Nursing Students’ Satisfaction and Self-Confidence After Short-Term Clinical Preparation: A Cross-Sectional Study

**DOI:** 10.3390/nursrep15090317

**Published:** 2025-09-01

**Authors:** Asim Abdullah Alhejaili, Bassam Alshahrani, Abdulrahman Muslihi, Paul Reinald Base Garcia, Mark Yuga Roque, Rawan Saud Alharbi, Hammad Ali Fadlalmola

**Affiliations:** 1 Department of Medical and Surgical Nursing, College of Nursing, Taibah University, Madinah 42353, Saudi Arabia; bshahrani@taibahu.edu.sa (B.A.); pgarcia@taibahu.edu.sa (P.R.B.G.); rsbharbi@taibahu.edu.sa (R.S.A.); 2 Honorary Fellow SMAH, School of Nursing, University of Wollongong, Wollongong 2522, Australia; 3 Department of Public Health, College of Nursing and Health Sciences, Jazan University, Jizan 45142, Saudi Arabia; amuslihi@jazanu.edu.sa; 4 Department of Maternity and Childhood, College of Nursing, Taibah University, Madinah 42353, Saudi Arabia; mroque@taibahu.edu.sa; 5 Department of Community Health Nursing, College of Nursing, Taibah University, Madinah 42353, Saudi Arabia; hafadlelmola@taibahu.edu.sa

**Keywords:** nursing education, clinical placement, preparatory courses, student satisfaction, self-confidence, Saudi Arabia

## Abstract

**Background/Objectives:** The transition from theoretical knowledge to clinical practice poses significant challenges for nursing students globally. This critical period requires comprehensive educational support to build confidence and competence. While short-term preparatory courses have shown promise internationally, their effectiveness within the Saudi Arabian context remains understudied. This study aimed to evaluate nursing students’ satisfaction and self-confidence following participation in short-term preparatory courses conducted before clinical placements at Taibah University, Saudi Arabia. **Methods:** A descriptive cross-sectional study was conducted from February to April 2025. Data were collected from 117 undergraduate nursing students (response rate: 80.7%) using a validated questionnaire adapted from the National League for Nursing’s Student Satisfaction and Self-Confidence in Learning instrument. The preparatory courses included nursing care plan development, hospital orientation, and infection control procedures delivered over two weeks. Statistical analysis included descriptive statistics and Pearson correlation analysis. **Results:** Students reported high levels of satisfaction (mean = 4.29 ± 0.92) and self-confidence (mean = 4.31 ± 0.81) scores. The highest satisfaction was with instructor effectiveness (mean = 4.31 ± 1.05) and teaching methods (mean = 4.32 ± 1.01). Students demonstrated strong confidence in personal learning responsibility (mean = 4.44 ± 0.88) and skill development (mean = 4.32 ± 0.95). A strong positive correlation existed between satisfaction and self-confidence (r = 0.79, *p* < 0.001). **Conclusions:** Short-term preparatory courses effectively enhanced nursing students’ satisfaction and self-confidence in the Saudi Arabian context. The strong correlation between these constructions suggests that educational interventions improving one dimension is likely to benefit the other. These findings support integrating structured preparatory programs into nursing curricula to facilitate successful clinical transitions.

## 1. Introduction

The transition from theoretical classroom learning to clinical practice represents one of the most challenging phases in nursing education globally [1]. This critical period, often characterized as the “theory-practice gap,” significantly impacts students’ confidence levels, professional identity formation, and clinical competence development [2]. Contemporary healthcare environments intensify these challenges, requiring students to navigate complex technical skills, interprofessional collaboration, ethical decision-making, and emotional demands while simultaneously meeting increasingly sophisticated competency standards [3].

The significance of this transition extends beyond individual student experiences to affect patient safety, quality of care, and healthcare workforce retention. Research indicates that inadequately prepared nursing students experience higher levels of anxiety, reduced clinical performance, and increased likelihood of early career attrition [4]. These challenges are particularly pronounced in rapidly evolving healthcare systems where technological advances, evidence-based practice requirements, and patient complexity continually reshape clinical expectations.

Extensive international research demonstrates the critical importance of structured preparatory courses in enhancing students’ readiness and confidence before clinical placement [5]. These interventions are particularly vital for developing competencies in nursing care plan formulation and infection control procedures—fundamental skills requiring seamless integration of theoretical knowledge with practical application [6]. Short-term intensive preparatory courses offer structured opportunities for skill development, theoretical concept reinforcement, and confidence building within controlled educational environments before students encounter real clinical settings [7].

### 1.1. Theoretical Framework

This study is grounded in Bandura’s self-efficacy theory, which posits that confidence in one’s abilities directly influences performance outcomes and learning effectiveness [8]. According to this theoretical framework, self-efficacy develops through four primary mechanisms: mastery experiences (successful performance of tasks), vicarious learning (observing others’ successful performances), verbal persuasion (encouragement and feedback), and physiological/emotional states (managing stress and anxiety). This theory provides a robust framework for understanding how structured preparatory courses can enhance students’ confidence through systematic practice opportunities, peer observation, instructor feedback, and stress management in supportive learning environments.

The application of self-efficacy theory to nursing education suggests that preparatory courses should incorporate multiple learning modalities to address all four sources of efficacy information. Mastery experiences through simulated practice, vicarious learning through demonstration and peer observation, verbal persuasion through constructive feedback, and emotional support through stress reduction strategies collectively contribute to enhanced self-confidence and subsequent clinical performance.

### 1.2. Literature Review

International evidence consistently demonstrates positive outcomes associated with pre-clinical preparatory programs across diverse educational contexts. A comprehensive systematic review by Cant and Cooper [9] analyzing 33 studies revealed that simulation-based preparatory courses significantly improve students’ clinical competence, critical thinking abilities, and self-efficacy levels. Their meta-analysis showed effect sizes ranging from moderate to large (Cohen’s d = 0.58–1.12) for confidence outcomes following structured preparatory interventions.

Recent studies have emphasized the importance of comprehensive preparatory approaches incorporating multiple learning strategies. Kim et al. [10] conducted a randomized controlled trial demonstrating that students participating in structured preparatory courses showed significantly improved clinical judgment (*p* < 0.001) and patient safety behaviors (*p* < 0.01) during subsequent clinical rotations compared to control groups. These findings support the value of intensive pre-clinical preparation in bridging the theory–practice gap.

The content and structure of preparatory courses significantly influence their effectiveness. Research by Lubbers and Rossman [11] found that programs incorporating active learning strategies, simulation experiences, and reflective practice components yielded superior outcomes compared to traditional didactic approaches. Their study of 127 nursing students revealed that those participating in multi-modal preparatory courses demonstrated 34% higher confidence scores and 28% better clinical performance ratings during initial clinical placements.

### 1.3. Saudi Arabian Context

Saudi Arabia’s healthcare sector has undergone substantial transformation aligned with Vision 2030 objectives, emphasizing the development of a highly skilled, internationally competitive nursing workforce [12]. This transformation has created unique opportunities and challenges for nursing education, including the need to integrate gender-specific educational environments, Islamic healthcare values, and multicultural clinical settings while maintaining international accreditation standards [13].

The Saudi nursing education landscape reflects broader societal changes, with increasing numbers of Saudi nationals entering the profession and expectations for world-class educational standards. Recent policy initiatives have emphasized evidence-based practice, technology integration, and alignment with international competency frameworks. These developments necessitate innovative approaches to clinical preparation that respect cultural values while ensuring graduates meet global standards.

Despite these significant developments, limited research has examined pre-clinical preparation effectiveness within the Saudi Arabian nursing education context. The unique cultural, linguistic, and educational factors characterizing this setting require context-specific evaluation of preparatory program outcomes. Understanding students’ perceptions and experiences within this distinctive environment is essential for evidence-based curriculum development and quality improvement initiatives that respect local values while achieving international standards.

### 1.4. Study Rationale and Objectives

This study addresses a significant gap in understanding preparatory course effectiveness within the Saudi Arabian nursing education context. While international literature provides substantial evidence supporting preparatory programs, the transferability of these findings to the Saudi context remains uncertain, given unique cultural, educational, and healthcare system characteristics. The findings aim to provide evidence-based insights for curriculum development, educational policy decisions, and quality improvement initiatives specific to Saudi nursing education while contributing to the broader international discourse on clinical preparation strategies.

Primary Objective: To evaluate nursing students’ satisfaction and self-confidence following participation in short-term preparatory courses prior to clinical placements at Taibah University, Saudi Arabia.

Secondary Objectives:

1. Assess satisfaction levels with instructional methodologies and learning materials utilized in preparatory courses;
2. Evaluate self-confidence in applying clinical skills following course completion;
3. Examine relationships between satisfaction and self-confidence variables;
4. Identify program strengths and areas for improvement from students’ perspectives.

## 2. Materials and Methods

### 2.1. Study Design

A descriptive cross-sectional design was employed to evaluate students’ perceptions immediately following preparatory-course completion. This methodological approach was selected for its appropriateness in assessing attitudes, perceptions, and experiences at a specific time point, providing valuable baseline data for educational practice and policy decisions [14]. The cross-sectional design enabled efficient data collection while capturing students’ immediate post-course impressions before clinical experiences potentially influenced their perceptions.

### 2.2. Setting and Context

The study was conducted at the College of Nursing, Taibah University, Madinah, Saudi Arabia, between February and April 2025. Taibah University serves as a leading educational institution in western Saudi Arabia, offering comprehensive nursing programs from bachelor’s to doctoral levels. The College of Nursing maintains strong partnerships with regional healthcare facilities, emphasizes evidence-based practice, and holds international accreditation from the Accreditation Commission for Education in Nursing (ACEN).

The preparatory courses comprised three integrated components delivered over a two-week intensive period immediately before clinical placement commencement. The first component, Nursing Care Plan Development, allocated 20 h focusing on systematic patient assessment, nursing diagnosis formulation using NANDA-I taxonomy, SMART goal setting, evidence-based intervention planning, and outcome evaluation. Students engaged in case study analysis, group discussions, and individual care plan development using standardized templates and electronic health record simulations.

The second component, Hospital Orientation and Professional Communication, comprised 15 h, emphasizing healthcare team collaboration, SBAR communication techniques, professional documentation standards, patient interaction skills, and cultural competence. Learning activities included role-playing scenarios, interprofessional simulation exercises, and communication workshops addressing common clinical situations.

The third component, Infection Control Procedures, provided 15 h of comprehensive coverage including standard precautions, transmission-based precautions, hand hygiene protocols, personal protective equipment usage, and environmental safety measures. Students participated in skills laboratories demonstrating proper techniques, completed competency assessments, and engaged in problem-based learning scenarios addressing infection prevention challenges.

### 2.3. Participants and Sampling

The target population consisted of Level 4 undergraduate nursing students enrolled in the Bachelor of Science in Nursing program. These students had completed three years of foundational theoretical courses, including anatomy, physiology, pharmacology, and nursing fundamentals, but had limited clinical experience (less than 100 h), making them ideal candidates for preparatory-course evaluation.

Inclusion criteria required current enrollment in Level 4 of the BSN program, successful completion of all prerequisite courses, participation in all three preparatory course components, and voluntary consent to participate in the study. Exclusion criteria encompassed previous clinical experience exceeding 100 h, incomplete preparatory course attendance (absent more than 10% of sessions), current employment in healthcare settings, and withdrawal of consent at any point during the study.

Convenience sampling was utilized due to practical considerations and the specific timing requirements of course completion. All eligible students (N = 145) were invited to participate through multiple communication channels, including institutional email, learning management system announcements, and classroom presentations by research team members not involved in course instruction.

### 2.4. Sample Size Determination

Sample size calculation utilized the Raosoft^®^ online calculator with parameters including a total population size of 145 eligible students, 95% confidence level, 5% margin of error, and 50% response distribution representing the most conservative estimate. The calculated minimum sample size was 106 participants. To account for potential non-response, incomplete questionnaires, and data quality issues, the target sample size was increased by 10%, resulting in a target of 117 participants, representing 80.7% of the eligible population.

### 2.5. Instrumentation

Data collection employed the “Nursing Student Satisfaction and Self-Confidence in Short-Term Course Learning” questionnaire, adapted from the validated National League for Nursing (NLN) “Student Satisfaction and Self-Confidence in Learning” instrument [15]. The original instrument has demonstrated strong psychometric properties across multiple nursing education contexts internationally, with reported Cronbach’s alpha values ranging from 0.85 to 0.94.

The adaptation process began with obtaining permission from NLN for instrument use and modification, followed by expert panel review involving three PhD-prepared nursing educators for content relevance assessment. The instrument underwent forward translation to Arabic by a certified translator, then back-translation to English by an independent translator. The bilingual nursing faculty reconciled the translations to ensure conceptual equivalence. Pilot testing with 15 students not included in the main study provided feedback for refinement, resulting in minor wording adjustments for cultural clarity.

The final questionnaire structure comprised three sections. Section A contained demographic information, including seven items addressing age, gender, marital status, previous clinical experience, academic performance (GPA), preferred learning style, and career intentions. Section B consisted of the satisfaction subscale, with five items measuring teaching-method effectiveness, learning-material variety, instructor quality, material motivation, and learning-style accommodation. Section C included the self-confidence subscale, with eight items assessing content mastery, critical content coverage, skill development, resource utilization, learning responsibility, help-seeking behavior, activity utilization, and instructor role perceptions.

All satisfaction and self-confidence items utilized 5-point Likert scales ranging from 1 (Strongly Disagree) to 5 (Strongly Agree), with higher scores indicating greater satisfaction or confidence levels.

### 2.6. Validity and Reliability

Content validity was established through a three-member expert panel comprising PhD-prepared nursing educators with clinical teaching experience, who assessed item relevance, clarity, and cultural appropriateness. Content Validity Index (CVI) calculations yielded item-level scores ranging from 0.83 to 1.00, with a scale-level CVI of 0.91, indicating excellent content validity according to established criteria [16].

Internal consistency reliability testing demonstrated strong psychometric properties. Cronbach’s alpha coefficients were calculated as 0.91 for the overall instrument, 0.89 for the satisfaction subscale, and 0.87 for the self-confidence subscale. These values exceed the 0.70 threshold for acceptable reliability and approach the 0.90 level, indicating excellent internal consistency [14].

### 2.7. Data Collection Procedures

Electronic questionnaires were distributed via Google Forms through institutional email addresses. This platform was selected for its security features, including SSL encryption, user accessibility, automatic data compilation capabilities, and compatibility with institutional systems. Students received survey invitations within 48 h of preparatory course completion to minimize recall bias while allowing time for reflection.

The data collection protocol commenced with an initial invitation email sent by a research coordinator not involved in teaching to avoid potential coercion. The survey link required a unique institutional login to ensure participant eligibility while maintaining anonymity. An information sheet was embedded before questionnaire access, clearly stating the estimated completion time of 10–15 min. A reminder email was sent after one week to non-respondents, followed by a second reminder three days before survey closure. The survey remained open for two weeks to maximize response rates while maintaining data collection within a reasonable timeframe.

Response restrictions prevented multiple submissions while maintaining anonymity through system-generated identifiers rather than personal information collection. This approach balanced data integrity needs with participant privacy protection.

### 2.8. Ethical Considerations

Ethical approval was obtained from the Research Ethics Committee, College of Nursing, Taibah University (Approval No. CON-REC-2025-05). This study adhered to the Declaration of Helsinki principles, local ethical guidelines for educational research, and international standards for research integrity.

Comprehensive ethical safeguards were implemented throughout the study. Informed-consent procedures included a clear explanation of voluntary participation and explicit statements that participation or non-participation would not affect academic standing. Data protection measures encompassed anonymization with removal of identifying information before analysis, secure storage on password-protected institutional servers, and limiting access to research team members. Plans for data destruction after five years aligned with institutional policy, and participants were offered the opportunity to receive aggregate results upon request.

### 2.9. Statistical Analysis

Data analysis utilized SPSS version 28.0 (IBM Corp., Armonk, NY, USA) with supplementary visualization using Python 3.9. The analytical approach incorporated both descriptive and inferential statistics appropriate for research questions and data characteristics.

Descriptive statistics included frequencies and percentages for categorical variables, along with means and standard deviations for continuous variables. Score interpretation guidelines classified results as high satisfaction/confidence for scores exceeding 4.0, moderate for scores between 3.0 and 3.99, and low for scores below 3.0. Inferential statistics employed a Pearson correlation analysis for examining satisfaction–confidence relationships, with correlation coefficient interpretation ranging from very weak (0.00–0.19), weak (0.20–0.39), moderate (0.40–0.59), strong (0.60–0.79), to very strong (0.80–1.00).

Data quality assurance procedures included normality assessment using Shapiro–Wilk tests, where *p* > 0.05 indicated normal distribution. Skewness and kurtosis evaluation confirmed acceptable ranges between −2 to +2. Missing-data analysis revealed less than 2% missing values, which were handled through listwise deletion. Outlier detection utilized standardized scores with z-scores exceeding ±3.29 investigated for potential data entry errors. The statistical significance threshold was set at *p* < 0.05 for all analyses.

## 3. Results

### 3.1. Response Rate and Demographic Characteristics

Of 145 eligible students, 117 completed the questionnaire (response rate: 80.7%). Table 1 presents participant demographic characteristics.

### 3.2. Student Satisfaction Outcomes

Analysis of the satisfaction subscale revealed consistently high scores across all domains (overall mean = 4.29 ± 0.92). Table 2 presents detailed satisfaction results.

### 3.3. Student Self-Confidence Outcomes

Self-confidence assessments demonstrated high overall scores (mean = 4.31 ± 0.81), with particularly strong performance in personal learning responsibility. Table 3 presents comprehensive self-confidence results.

### 3.4. Correlation Analysis

The Pearson correlation analysis revealed a strong positive relationship between student satisfaction and self-confidence (r = 0.79, *p* < 0.001). Data normality was confirmed through Shapiro–Wilk testing, supporting the use of parametric correlation analysis. Table 4 presents correlation matrix for primary variables.

## 4. Discussion

### 4.1. Principal Findings

This study provides empirical evidence regarding nursing students’ satisfaction and self-confidence following short-term preparatory courses within the Saudi Arabian educational context. The consistently high levels of both satisfaction (mean = 4.29 ± 0.92) and self-confidence (mean = 4.31 ± 0.81) demonstrate the effectiveness of structured preparatory interventions in enhancing student readiness for clinical practice. The strong positive correlation between these constructions (r = 0.79, *p* < 0.001) suggests their interdependent nature and mutual reinforcement.

### 4.2. Interpretation of Satisfaction Outcomes

The high satisfaction scores across all measured domains indicate successful course design and implementation aligned with students’ learning needs. Students particularly valued teaching-method effectiveness and instructor quality, with over 84% expressing agreement or strong agreement in these areas. These findings corroborate previous research emphasizing the critical importance of the pedagogical approach and instructor competence in nursing-education outcomes [11].

The consistency of satisfaction scores across different course components suggests comprehensive program quality rather than isolated areas of excellence. This holistic satisfaction is particularly important given the integrated nature of clinical practice, where students must synthesize knowledge and skills from multiple domains. The variety in learning materials and activities (mean = 4.26) indicates successful accommodation of diverse learning preferences, essential for adult learners with varying educational backgrounds and experiences.

### 4.3. Self-Confidence Development

Students demonstrated substantial confidence across all measured competencies, with a particularly strong performance in personal learning responsibility (mean = 4.44 ± 0.88). This finding holds significant implications, as self-directed learning capabilities are essential for successful clinical practice and lifelong professional development [17]. The development of autonomous learning skills during preparatory courses may facilitate smoother transitions to the independent decision-making required in clinical settings.

The high confidence scores for content mastery and skill development suggest effective preparation for the transition from theoretical learning to clinical application. Students’ confidence in their ability to utilize course activities for skill acquisition (mean = 4.39) indicates successful metacognitive development—an awareness of how to learn effectively that extends beyond specific content knowledge.

### 4.4. Theoretical Implications

The strong positive correlation between satisfaction and self-confidence provides empirical support for Bandura’s self-efficacy theory within the nursing-education context [8]. This relationship suggests that positive learning experiences (reflected in satisfaction scores) contribute to enhanced self-efficacy beliefs (measured through confidence scores). The bidirectional nature of this relationship implies that interventions targeting either construction may yield benefits for both.

The findings also support constructivist learning principles, where active engagement, social interaction, and authentic learning experiences contribute to knowledge construction and confidence development. The preparatory courses’ emphasis on practical skill development, peer collaboration, and instructor support aligns with these theoretical foundations.

### 4.5. Cultural and Contextual Considerations

The Saudi Arabian educational context presents unique considerations that may have influenced study outcomes. The gender-segregated learning environment, while culturally mandated, may have contributed to enhanced comfort and participation levels, particularly among female students who comprised most participants. This segregation potentially reduces social anxiety and facilitates more open communication during skill practice sessions [18].

The integration of Islamic values in healthcare education and emphasis on collaborative learning aligned well with the preparatory course methodologies. The concept of collective responsibility and mutual support inherent in Islamic teachings may have enhanced peer learning and group cohesion during preparatory activities. Additionally, the respect for instructor authority within Saudi culture may have contributed to high satisfaction scores related to teaching effectiveness.

The multicultural nature of Saudi healthcare settings, with professionals from diverse international backgrounds, underscores the importance of preparatory courses in developing communication skills and cultural competence. Students’ high confidence in these areas suggests successful preparation for the linguistic and cultural diversity they will encounter in clinical practice.

### 4.6. Implications for Nursing Education

These findings have several important implications for nursing-education practice and policy development:

Curriculum Integration: The results provide strong empirical support for mandating intensive, focused preparatory courses as integral curriculum components rather than optional activities. The two-week intensive format appears optimal for knowledge consolidation and skill development without causing learner fatigue.

Faculty Development: The importance of instructor effectiveness highlights the need for careful faculty selection and ongoing professional development for preparatory program delivery. Institutions should invest in training faculty members in active learning strategies, simulation facilitation, and formative assessment techniques.

Resource Allocation: The success of diverse teaching methods suggests that nursing programs should allocate sufficient resources for simulation laboratories, case study development, and interactive learning technologies. The return on investment appears justified given the enhanced student outcomes.

Assessment Strategies: The high self-confidence scores suggest that preparatory courses successfully address affective learning domains often neglected in traditional assessment approaches. Programs should incorporate confidence assessment alongside knowledge and skill evaluation.

### 4.7. Recommendations for Practice

Based on study findings, several evidence-based recommendations emerge for enhancing preparatory course effectiveness. The two-week intensive structure appears optimal for focused skill development and should be preserved rather than distributing content across longer periods, as this concentrated format facilitates deep learning and skill consolidation. Programs should continue prioritizing interactive teaching methods, simulation experiences, and collaborative learning activities that contribute to high satisfaction scores, as these pedagogical approaches align with adult learning principles and contemporary educational best practices.

Institutions should explicitly teach metacognitive strategies and self-assessment techniques to further enhance students’ learning autonomy, recognizing that self-directed learning capabilities are essential for lifelong professional development. Rigorous faculty selection criteria should be implemented alongside ongoing professional development in clinical teaching methodologies, ensuring instructors possess both the content expertise and pedagogical skills necessary for effective preparatory course delivery. Finally, systematic evaluation processes should be established to monitor program effectiveness and implement continuous quality improvements, utilizing both quantitative metrics and qualitative feedback to inform evidence-based program modifications.

## 5. Conclusions

This study provides strong empirical evidence supporting the effectiveness of structured short-term preparatory courses in enhancing nursing students’ satisfaction and self-confidence within the Saudi Arabian educational context. The consistently high levels of satisfaction and self-confidence, coupled with their strong positive correlation, demonstrate the value of comprehensive pre-clinical preparation programs.

For educators and policymakers, the results emphasize the importance of investing in well-designed preparatory programs incorporating diverse teaching strategies, skilled instructors, and comprehensive content addressing both technical skills and professional development.

Future research should extend these findings through longitudinal designs, objective performance measures, and cross-cultural comparisons to further strengthen the evidence base for optimal preparatory course design and implementation. The ultimate goal remains the preparation of nursing graduates who are not only technically competent but also confident and satisfied with their educational preparation for professional practice.

## Figures and Tables

**Table 1:** Participant demographic characteristics (N = 117).

Characteristic	Category	Frequency (*n*)	Percentage (%)
Age	19–20 years	42	35.9
	21–22 years	62	53.0
	23–24 years	13	11.1
Gender	Female	86	73.5
	Male	31	26.5
Marital Status	Single	107	91.5
	Married	10	8.5
Previous Clinical Experience	None	45	38.5
	<50 h	34	29.1
	50–100 h	38	32.5
Academic Performance (GPA)	3.50–4.00	72	61.5
	3.00–3.49	35	29.9
	2.50–2.99	10	8.5

**Table 2:** Student satisfaction with short-term preparatory courses (N = 117).

Statement	Mean (±SD)	Strongly Disagree *n* (%)	Disagree *n* (%)	Undecided *n* (%)	Agree *n* (%)	Strongly Agree *n* (%)
The teaching methods were helpful and effective	4.32 (±1.01)	5 (4.3)	4 (3.4)	9 (7.7)	26 (22.2)	73 (62.4)
The course provided variety in learning materials and activities	4.26 (±1.00)	4 (3.4)	6 (5.1)	9 (7.7)	33 (28.2)	65 (55.6)
I enjoyed how my instructor taught the topics	4.31 (±1.05)	6 (5.1)	4 (3.4)	7 (6.0)	29 (24.8)	71 (60.7)
The teaching materials were motivating and helped me learn	4.27 (±1.05)	6 (5.1)	4 (3.4)	7 (6.0)	29 (24.8)	71 (60.7)
The instructor’s teaching style suited my learning preferences	4.29 (±1.03)	5 (4.3)	5 (4.3)	7 (6.0)	30 (25.6)	70 (59.8)
Overall Satisfaction Score	4.29 (±0.92)					

**Table 3:** Student self-confidence following preparatory courses (N = 117).

Statement	Mean (±SD)	Strongly Disagree *n* (%)	Disagree *n* (%)	Undecided *n* (%)	Agree *n* (%)	Strongly Agree *n* (%)
I am confident that I am mastering the content	4.26 (±1.04)	6 (5.1)	4 (3.4)	8 (6.8)	31 (26.5)	68 (58.1)
I am confident the course covered critical content for nursing skills	4.31 (±0.97)	4 (3.4)	4 (3.4)	8 (6.8)	32 (27.4)	69 (59.0)
I am confident I am developing skills for clinical settings	4.32 (±0.95)	4 (3.4)	3 (2.6)	8 (6.8)	33 (28.2)	69 (59.0)
My instructors used helpful resources	4.29 (±0.97)	4 (3.4)	4 (3.4)	9 (7.7)	32 (27.4)	68 (58.1)
It is my responsibility as a student to learn	4.44 (±0.88)	3 (2.6)	3 (2.6)	5 (4.3)	28 (23.9)	78 (66.7)
I know how to get help when I need it	4.30 (±0.92)	3 (2.6)	4 (3.4)	8 (6.8)	35 (29.9)	67 (57.3)
I know how to use course activities to learn critical skills	4.39 (±0.96)	4 (3.4)	4 (3.4)	6 (5.1)	27 (23.1)	76 (65.0)
It is the instructor’s responsibility to tell me what to learn	4.22 (±1.12)	7 (6.0)	5 (4.3)	11 (9.4)	25 (21.4)	69 (59.0)
Overall Self-Confidence Score	4.31 (±0.81)					

**Table 4:** Correlation matrix for primary variables.

Variable	1	2	Mean	SD
1. Overall Satisfaction	1.00		4.29	0.92
2. Overall Self-Confidence	0.79 **	1.00	4.31	0.81

Note: ** indicates a statistically significant correlation at *p* < 0.001.

## Data Availability

The data presented in this study are available upon reasonable request from the corresponding author. The data are not publicly available due to privacy and ethical considerations involving student information.

## Public Involvement Statement

No public involvement in any aspect of this research.

## Guidelines and Standards Statement

This manuscript was drafted against the STROBE (Strengthening the Reporting of Observational Studies in Epidemiology) guidelines for cross-sectional studies research.
